# Endogenous Endophthalmitis Associated with Liver Abscess Successfully Treated with Vitrectomy and Intravitreal Empirical Antibiotics Injections

**DOI:** 10.1155/2020/8165216

**Published:** 2020-07-03

**Authors:** Sung Woong Lim, Youngje Sung, Hee Jung Kwon, Won Kyung Song

**Affiliations:** Department of Ophthalmology, CHA Bundang Medical Center, CHA University College of Medicine, Seoul, Republic of Korea

## Abstract

**Purpose:**

Klebsiella pneumoniae is the most common pathogen of endogenous endophthalmitis accompanying liver abscess in East Asia. The treatment may be different for the patients with endogenous endophthalmitis from the postoperative endophthalmitis. Prompt administration of both intraocular (vancomycin and ceftazidime) and systemic (ceftriaxone, aminoglycoside, and/or metronidazole) antibiotics have been a mainstay of treatment. However, ceftriaxone has been proven to more effectively kill K. pneumoniae than ceftazidime in in vitro studies, and the safety of intravitreal ceftriaxone has been confirmed in animal studies.

**Methods:**

Two diabetic female patients with liver abscess presented with decreased visual acuity of the unilateral eyes. Fundus photography, ocular ultrasonography, and abdominal computed tomography were performed.

**Results:**

A 50-year-old diabetic female patient with liver abscess presented decreased visual acuity of the left eye. In fundus examinations, a yellowish necrotic lesion was noted throughout the eye. The results of culture of the blood culture was positive for K. pneumoniae. She was successfully treated with intravitreal ceftazidime injections, and the remaining vitreous opacity was treated with vitrectomy. A 62-year-old female with liver abscess presented a visual symptom of floaters in the right eye. The fundus had a hazy appearance through the vitreous opacity. A yellowish-white subretinal abscess was noted at the temporal macula. Cultures of blood were negative. She underwent intravitreal injections of empirical antibiotics. However, she did not respond to intravitreal vancomycin and ceftazidime. Thus, we changed the intravitreal antibiotics from ceftazidime to ceftriaxone and performed vitrectomy. Her ocular status significantly improved after this change.

**Conclusion:**

Our results indicate that for cases with EE, prompt initial treatment with broad spectrum antibiotics, followed by rapid use of antibiotics selected according to culture results, and empirical use of antibiotics in cases of a negative culture may be an effective treatment. Vitrectomy also can be an effective treatment option for vitreous opacity refractory to the treatment.

## 1. Case Report

The prognosis of patients who develop endogenous endophthalmitis (EE) is devastating despite aggressive therapy, with many cases resulting in irreversible vision loss [1]. We report EE cases in which intravitreal antibiotic treatment saved the patients from visual losses.

**Figure 1:**
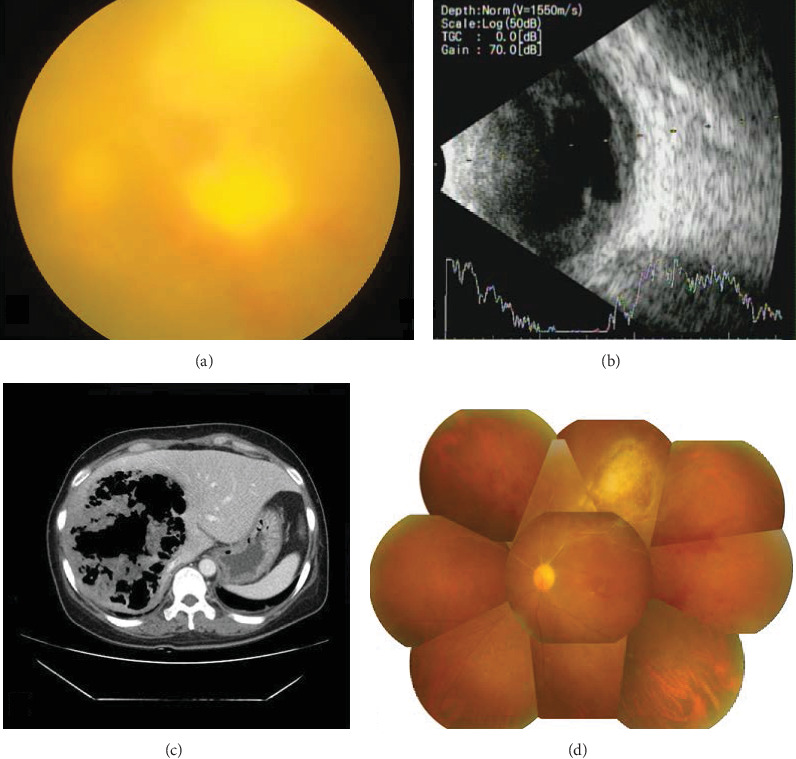
(a) Funduscopy shows a large necrotic lesion in the macular and superotemporal area with vitreous opacity of the left eye. (b) Ultrasound shows opacity and a diffuse abscess formation. (c) Abdominal computed tomography shows a gas-forming pyogenic liver abscess of the right lobe. (d) Fundus after 1 month of surgery, showing an atrophic lesion in the superotemporal area, and retinal hemorrhage around the macula.

A 50-year-old female was referred under suspicion of hepatitis because of vomiting and jaundice symptoms for 2 weeks. Her only medical history was uncontrolled diabetes mellitus. A computed tomography scan of the abdomen showed a 16 cm gas-forming abscess in the right lobe and left medial segment of the liver (Figure 1(c)). Intravenous administration of antibiotics (ceftriaxone, metronidazole, and amikacin) was started, and emergent percutaneous drainage was performed for the liver abscess. On the fourth day of admission, the patient was referred to the ophthalmology clinic because of decreased visual acuity in the left eye. The best-corrected visual acuity (BCVA) was hand motion in the left eye. In slit lamp examinations, many cells and a 3 mm high hypopyon were observed in the anterior chamber of the left eye. In fundus examinations, the optic disc was not visualized, and a yellowish necrotic lesion was noted throughout the eye (Figure 1).

She underwent intravitreal vancomycin (1 mg/0.1 mL) and ceftazidime (2.5 mg/0.1 mL) injections on the fourth day of admission. She was also prescribed topical antibiotics (fortified cefazolin, fortified tobramycin, and moxifloxacin), as well as treatment with corticosteroids and anticholinergics. The results of culture of the intra-abdominal abscess cavity and blood culture were positive for *Klebsiella pneumoniae* (negative for extended spectrum beta-lactamase (ESBL)) on the third day of admission. This strain was resistant to ampicillin only but was susceptible to all other antibiotics. Intravitreal injections of ceftazidime without vancomycin were given nine times, and dexamethasone was given four times every 3–4 days. Despite several intravitreal injections, vitreous opacity remained. Pars plana vitrectomy (PPV) and an intravitreal ceftazidime (2.5 mg/0.1 mL) injection were performed in the left eye 30 days after admission. An intraoperative superotemporal subretinal abscess was noted. On postoperative day 39, a fundus examination showed a resolved abscess and a decreased whitish lesion. On the last follow-up at 18 months, stable atrophy at the location of the retinal abscess area was noted and best-corrected visual acuity was 0.08 (Figure 1(d)).

**Figure 2:**
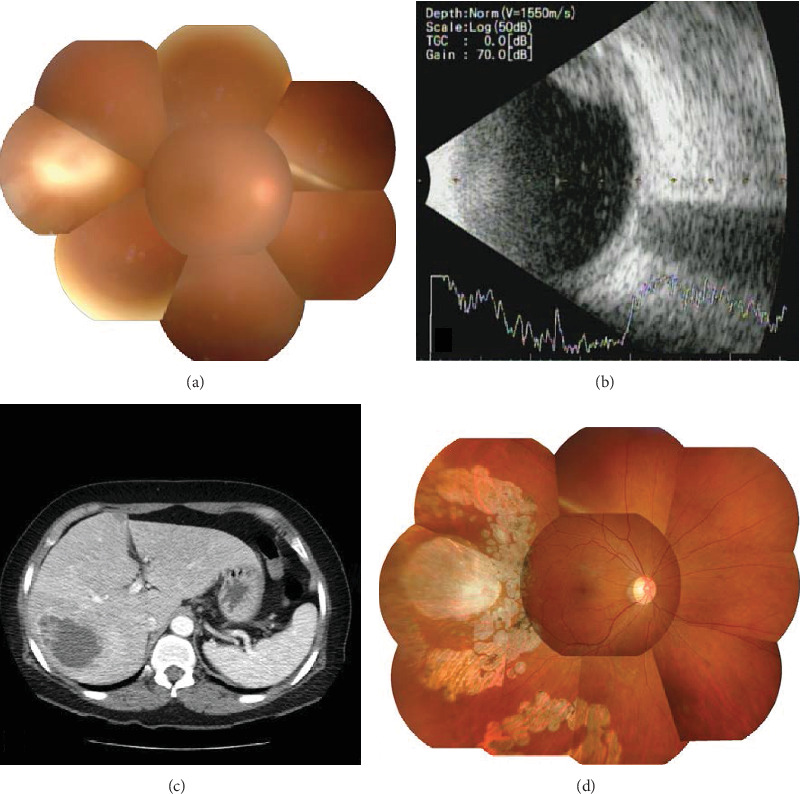
(a) Funduscopy shows a large necrotic lesion in the superotemporal area with vitreous opacity of the right eye. (b) Ultrasound shows abscess formation with a height of 5 mm at the temporal macula (c) Abdominal computed tomography shows a pyogenic liver abscess of the right lobe. (d) Fundus, 18 months after admission showing a resolved abscess and atrophic retina at the original abscess site.

A 62-year-old female presented with a 3-day history of chilling, diarrhea, and a visual symptom of floaters in the right eye. The initial BCVA was counting fingers in the right eye, 0.63 in the left eye, and a normal intraocular pressure. Slit lamp examination revealed ciliary injection as well as subconjunctival hemorrhage and many cells with flare in the anterior chamber of the right eye. The fundus had a hazy appearance through the vitreous opacity. A yellowish-white, round, elevated lesion three times the diameter of the optic disc was noted at the temporal macula (Figure 2(a)). Ultrasonography revealed multiple vitreous opacities and an elevated lesion with a height of 5 mm at the posterior pole, consistent with a subretinal abscess (Figure 2(b)). In a computed tomography scan of the abdomen, a pyogenic liver abscess was detected in segments VII/VIII (Figure 2(c)).

She was initially treated with empirical antibiotics (systemic moxifloxacin). She also underwent intravitreal injections of antibiotics (1 mg/0.1 mL vancomycin, 2.5 mg/0.1 mL ceftazidime, and 400 *μ*g/0.1 mL dexamethasone) on the initial visit day. She was admitted to the internal medicine department, and the liver abscess was drained with pigtail catheterization. Systemic antibiotics were changed to ceftriaxone, amikacin, and metronidazole according to the recommendation of a gastroenterology specialist. The results of cultures of blood and drained fluid on the initial day of admission were negative. The results of vitreous and anterior chamber cultures on the initial day were negative on the fourth day after admission. Cultures of blood and the liver abscess were also negative. On the third day, the visual acuity of the right eye worsened to hand motions and the vitreous turbidity became denser. She underwent PPV and intravitreal injection. The antibiotics were changed to 2 mg/0.1 mL ceftriaxone from ceftazidime and vancomycin to treat Gram-negative bacteria, the most common pathogens of metastatic endophthalmitis from a liver abscess. Although the vitreous opacity was alleviated dramatically after the vitrectomy, subretinal abscess was remained. She underwent intravitreal ceftriaxone and dexamethasone injection again on the fourth day after the vitrectomy. Vitreous opacity and anterior chamber reactions improved after 1 day, and the size of subretinal abscess was dramatically improved 2 days after the second intravitreal injection. Fourteen days after the second intravitreal injection, the subretinal abscess was completely resolved and the BCVA had improved to 0.2. She was discharged. At the 40-month follow-up, the BCVA of the right eye was 0.5 and a stable atrophic lesion was noted at the previous retinal abscess area (Figure 2(d)).

*Klebsiella pneumoniae*, the most common pathogen of EE accompanying liver abscess in east Asia, is associated with poor visual outcomes [2, 3]. It is very destructive, with a rapid progression, so prompt use of empirical antibiotics is the most effective treatment. In contrast to postoperative endophthalmitis, there are presently no clear guidelines on the management of EE, in particular, on the selection of systemic antibiotics and the role of vitrectomy in its management [4]. Ceftazidime and amikacin were usually selected as the initial antibiotics according to the Endophthalmitis Vitrectomy Study (EVS) protocol. However, the treatment may be different for the patients with EE because EVS protocol was established for the patients with postoperative endophthalmitis. Lee et al. [4] recommended ceftriaxone for EE complicated with liver abscess as initial antibiotics in Korea, considering the regional variations in the causative pathogens in East Asia. They also reported that early vitrectomy (within 10 days after the presentation) may be helpful in patients with a visual acuity better than hand motion. In *in vitro* studies, ceftriaxone has been proven to more effectively kill *K*. *pneumoniae* than ceftazidime [5–8], and the safety of intravitreal ceftriaxone has been confirmed in animal studies [9]. The second patient did not respond to intravitreal vancomycin and ceftazidime, suggesting resistance to the antibiotics. Thus, we performed vitrectomy and changed the intravitreal antibiotics from ceftazidime to ceftriaxone. Her ocular status was dramatically improved after this change.

Taken together, our results indicate that for cases with EE, rapid use of antibiotics was selected according to culture results, and empirical use of antibiotics in cases of a negative culture may be an effective treatment. Vitrectomy also can be an effective treatment option for vitreous opacity refractory to the treatment.

## Data Availability

The data that support the findings of this study are available from the corresponding author upon reasonable request.
